# Pulsating Tumors

**Published:** 1828-04

**Authors:** 


### PULSATING TUMORS.

Case I. (condensed from the *Medico-Chirurgical Review.*)

J. Nowlan, æt. twenty-two, was admitted into the Institution in Panton-square, with a pulsating swelling on the left parietal bone, in the situation of the posterior temporal artery. It could be emptied by pressure, but a large communicating branch passed across the vertex from the opposite temple, and contributed to supply it with blood. The cranium beneath appeared to be very considerably absorbed. The temporal artery had been secured (at least it was so supposed) by Mr. Babington, but with no effect. The day after the man's admission, the common carotid artery was tied by Mr. Wardrop, in order, as the reporter tells us, "to completely arrest from almost every channel, the supply of blood to this rapidly increasing vascular tumor." Much difficulty was experienced in getting the needle round the artery, and a free bleeding took place (so goes the report) from two large thyroideal veins. The pulsation ceased, but the tumor did not collapse, and next day a thrill was again perceptible. On the fifth day, there was much fever, and he was bled to syncope. On the seventh, hemorrhage, partly venous and partly arterial, occurred to a considerable extent, and he was again bled to fainting. On the ninth day, he was once more largely bled. Venous hemorrhage had occurred daily since the operation; the wound was not disposed to unite, and the tumor distinctly pulsated.

Eleventh day.—Pain in the left eyeball and orbit, with deafness, drowsiness, and mental disturbance, which increased on the 12th almost to coma, with contracted pupil.

On the thirteenth day, he was better, but the eyeball was found to be greatly protruded. The protrusion in the eyeball, however, continued to increase; the sclerotica sloughed, and the humors were discharged. The ligature came away on the twenty-fifth, and the wound quickly healed.

Nothing further was heard about the case until the 4th January, when the poor fellow was admitted into the Middlesex Hospital, with lumbar abscess, and altogether in a most miserable state. On the 21st he died. The tumor at this time was large, and pulsated very strongly.

On dissection, the posterior temporal artery was found to divide into two branches,—one running, as usual, beneath the skin; the other perforating the tendon of the occipito-frontalis, and then, considerably enlarged, twisting and turning and coiling upon itself, looking, when injected, as like a varicose vein as it is possible for a vessel which has no valves to resemble one which has them. On examining the neck, the common carotid was obliterated, and converted into a cellular cord just up to its bifurcation, whilst the jugular vein was in nearly the same state of degeneration from the site of the ligature up to the division of the carotids, from which to the base of the brain it was plugged with coagulum. There was much pus at the basis cranii, and around the commissura tractuum opticorum; it lay between the pia mater and arachnoid, and was continued within these membranes down the whole length of the spinal cord. The cranium was somewhat grooved beneath the tumor, just as it would be beneath a powerful muscle, but the pericranium was entire, and the inner surface of the bone was perfectly smooth and equal.

---

Case II.—W. Maclure, æt. thirty, a discharged soldier, applied to Dr. Maclachlan, on the 9th of July, 1825. The following is the account of the symptoms:

"Having caused that side of the head to be shaved, the better to observe the nature of the tumor, it presented the following appearances:—Soft, puffy, pulsating, and somewhat elastic swellings, of a varicose appearance, were found to occupy the course of the temporal, posterior auris, and occipital arteries, and their principal branches, each branch terminating by a tortuous extremity. These swellings could be made partly to disappear on pressure, but on its removal they speedily regained their former violence. They pulsated throughout their whole extent, and the pulsations were synchronous with those of the heart. By pressing on the common carotid, the pulsations ceased all along the swellings; and, by intercepting the flow of blood through the temporal or posterior auris, the throb was interrupted in corresponding parts of the tumor. They were not painful on being handled, but he complained much of the torture he had experienced for the last two months, from the throbbing, which often deprived him of rest for nights together, and, as he said, made his existence miserable to him. The integuments covering the swellings were of their natural colour; only at those points which were most prominent they had a slightly bluish-red tinge.

"This arborescent tumor commenced in front of the ear, immediately over the zygoma, and quickly swelling out, it became of the size of a split lemon, lying transversely over the ear. It sent a process forwards on the forehead, communicating by a tortuous extremity with the supraorbitary twig from the internal carotid; a large process upwards to the crown of the head; and backwards, the main body of the tumor communicated with the puffy swellings of the posterior auris and occipitalis, which latter vessels gave a varicose feeling to the scalp over the left side of the occiput.

"The largest and most prominent part of the tumor was immediately over the ear: at this point the throbbing was very violent, and the integuments being very thin and rather pointing, it threatened ere long to burst.

"The history he gave of his disease was the following: about ten years previously he had the temporal artery opened for an attack of ophthalmia. A small aneurismal tumor formed at the point of incision, for the cure of which the artery was cut across, lower down; but this not succeeding, the vessel was again exposed and a ligature applied. The little tumor disappeared, he says, only for a time: on its return, it was but small, gave him no uneasiness, and, although he served as a soldier for five years afterwards, he never complained of it to his surgeon.

"This disease seemed to me different from any kind of aneurism by anastomosis that I had either seen or read of. It evidently followed the ramifications of particular arteries; for, by pressure being made on a particular vessel, a corresponding portion of the tumor became flaccid and pulseless, showing distinctly that no free intercourse by means of cells existed in it.

"I proposed the trial of pressure: he said it had already been employed, and that it gave him so much pain that he would not again submit to it. He urged me at once to proceed to tie the carotid artery, as he was informed that that was the only means by which the disease could be effectually cured. I then explained to him the possibility of taking up the vessels singly; and that, should we fail, it was then in our power to tie the common carotid. He agreed; and, with the assistance of Professor Towers and Dr. Anderson, I began by laying bare the temporal artery as it emerges from the parotid gland: but, on dividing the fascia-like substance which kept it in situ, it shot forth through the opening in the form of a loop, in calibre larger than a goose-quill, thinner in its coats, and, if possible, more diaphanous than a vein; and thrilling violently at each pulsation. A ligature was applied to this loop; the wound was brought together with adhesive plaster, and, for additional security, a firm compress and bandage were applied. It was now evident that the vessel was diseased at the point of ligature, and the propriety of tying the common carotid hence became obvious. Pulsation had, however, ceased in the anterior and central portions of the tumor, which felt flaccid and doughy, showing that this plan of treatment would, in all probability, have been successful, so far as the vessels of the scalp, at least, were concerned, could the state of the arteries have been trusted to.

"Next day the common carotid was tied, in the presence and with the concurrence of Professors Burns and Towers, Drs. King and Anderson. An incision, about two inches and a half in length, was made along the inner edge of the sterno-cleido-mastoid muscle, commencing at the lower edge of the thyroid cartilage, and extending downward to within half an inch of the sternal extremity of the clavicle. A large branch of the exterior jugular vein ran across the line of incision, but, by carefully cutting through the fascia-like platysma myoides, the vessel was easily drawn aside along with that muscle. The sterno-cleido-mastoideus was now seen forming the outer margin of the wound, and the omo-hyoideus crossing it superiorly. The dissection was cautiously carried deeper, until the descendens noni was seen over the sheath of the vessels. The artery and par vagum were now distinctly in view, but the internal jugular vein, which frequently gives much trouble during this operation, did not at all appear. The sheath was opened by cautiously scratching with the point of the scissors between the carotid and par vagum, which nerve was carefully drawn aside. A blunt aneurismal needle, armed with a very fine silk ligature, was now introduced, and passed with ease, from without inwards, under the vessel. The ligature being tied, the ends were cut short, and the artery left undivided. The wound was brought together with sticking plaster, and a light compress and bandage applied. He did not lose above a spoonful of blood; and his pulse, immediately after the operation, was seventy-eight, and of good strength. Immediately on tying the vessel, the varicose tumors of the head became devoid of pulsation and felt flaccid, although their prominence was but little diminished.

"In the evening he felt his neck rather stiff; had slight headache, seated principally under the right temple. Took some food with relish. Pulse eighty-four; skin rather hot.

He gradually sunk, and died on the 14th.

"*Dissection*, fifty hours after death, in presence of Dr. Anderson and several medical gentlemen of Paisley, to which town the body had been removed. The weather being very hot, putrefaction had made considerable progress. The viscera of the abdomen appeared healthy. The intestines were much distended with flatus, devoid of fæces, and blanched.

"In the chest, some straw coloured puriform matter was found in the anterior mediastinum: about a pint of thick, greyish, muco-purulent matter in the right cavity of the pleura, and a small quantity of bloody extravasation into the left. The pericardium was unusually devoid of fluid; the heart large and flaccid.

"The wound, which had adhered throughout its whole extent by the first intention, had partially reopened from putrefaction. The carotid, the par vagum, and jugular vein appeared as if they never had been disturbed, nor was there the least appearance of pus around the ligature. On slitting up the artery, and cutting through the ligature at the same time, (the ligatured portion having been previously removed from the body,) small but soft clots were found above and below the ligature, and the artery remained puckered from the recent deposition of lymph. Its inner coats were divided as with a knife, while its external was found dense, strong, and entire. Below the ligature, the inner coat of the artery was of a vermilion red colour; even that of the thoracic aorta bore equal marks of inflammation, but at the bifurcation it was of its natural aspect; that portion also of the aortic arch nearer the heart than the coming-off of the left carotid was healthy. The carotid in the neck was of its usual size, strength, and thickness; but, on examining its branches on the head, they were found to have degenerated into dilated tubes of extreme thinness and transparency; which apparently yielding to the impetus of the blood, had become elongated, contorted, and ultimately convoluted on themselves, so as to form, by this species of doubling, the tumors which constituted this singular disease. These tumors felt like the placenta, and to the eye the larger portion immediately over the ear looked precisely like a bundle of earth-worms coiled together.

"I regret that, from the peculiar circumstances under which this inspection was obtained, I had not an opportunity of examining, more at leisure, this very unusual disease of the arterial system, or even of ascertaining the exact point at which the disorganization commenced; whether the artery became gradually thinner, or whether the disease began suddenly; whether in the diseased portions the three coats existed, or the dense but thin external one only remained; whether the branches of the internal carotid were similarly affected with the external, and thus giving rise to the epileptic fits to which he had recently become subject. These points unfortunately must be left to conjecture.

"I have been at some pains in searching through books for analogous cases, but PELLETAN is the only author, as far as I know, who has distinctly described this disease. In his Clinique Chirurgicale, tome ii. two cases are given, which coincide in every particular with the one here detailed. One of these only, a girl eighteen years of age, he had an opportunity of treating. Compression was first tried, but the patient could not bear it. He then tied the temporal artery: this promised to be successful, as far as the portion of the tumor supplied by that vessel was concerned, when unexpectedly the patient died in consequence of an 'indigestion.' He has fortunately favoured us with plates of this case, which are of great assistance in elucidating this subject, for he has said but little pathologically of the nature of the disease. He speaks merely of dilatation, but it is evident, from plate ii. fig. 2, in which the convolutions of the arteries of the scalp are given, on dissection, that the view above advanced, viz. the doubling of the dilated vessel on itself, as the cause of the tumor, is a correct one. BOYER,* also, who saw this case, and has given the dissection more at length, says, in speaking of the structure of the tumor,—"Toutes les artères comprises dans la tumeur, au dessous du tissu donc nous venons de parler, étaient dilatées, flexueuses, bosselées, ici très larges, là très étroites, pleines de sang caillé, ou d'une humeur blanche et épaisse. L'artère temporale depuis son origine, jusque vers le milieu de la tempe, avait éprouvé une simple dilatation.—Plus haut l'artère temporale, et ses diverses branches, étaient bosselées, flexueuses, grossés, et rouges." His remarks on the occipital branches are to the same effect.

"Mr. JOHN BELL describes aneurism by anastomosis, to which the disease under consideration has most affinity, to be 'a congeries of active arteries, absorbing veins, and intermediate cells.' Now, in the tumor which I have attempted to describe, there were no cells, no parenchyma, as in the spleen; the bulk of the tumor was formed almost entirely by convoluted dilated arterial *trunks*, the veins being but little changed from their healthy state. These arteries did not appear to communicate more freely than by their ordinary inosculations; and, in the less prominent parts of the swelling, they had more of the appearance of the contorted vessels of the gravid uterus, as represented in TIEDEMANN's beau-

* Traité des Mal. Chir. tome ii. p. 295.

tiful plate, than any other anatomical comparison I can give. Mr. Abernethy* evidently alludes to this disease when he says, while speaking of nævus, 'For this preternatural enlargement of vessels is not always cutaneous. I have seen it occupying the whole substance of the cheek, neither appearing beneath the skin nor the membrane of the mouth. I have seen it in the orbit,' &c. The cases reported by Messrs. Travers and Dalrymple, in the London Medico-Chirurgical Transactions, vol. ii. and vi., in which the carotid artery was tied for pulsating tumors of the orbit, appear to have been of this description.

"I have made these remarks and extracts because I conceive there exists a pulsating tumor composed entirely of dilated and convoluted arteries, whose inosculations and interlacements are not more numerous than usual, only they become more apparent from their increased size. That this tumor may occupy a great extent of surface, such as the side of the head, the neck, or arm, from its following in a continuous manner the course of the arteries. That the term aneurism by anastomosis is not very applicable to it; and that, from the advanced age of many of the persons in whom this disease has been met with, its congenital nature is more than doubtful, although Boyer, Pelletan, and others, are of this opinion. Whether, in the case of Maclure, the vessels were perfectly sound at the period of opening the temporal artery, may admit a doubt. The difficulty of curing the small aneurismal tumor favours this doubt: yet there certainly existed no obvious trace of enlargement of these vessels to lead to this conclusion. In explanation of this remark, it may be necessary to state that the artery was opened by myself. The opening of the temporal artery, however, probably operated as an exciting cause on vessels already disposed to disease."

The above case is taken from the *Glasgow Medical Journal*, conducted by Mr. Mackenzie, with whose valuable papers on the Eye the readers of this Journal must be familiar.

---

### ANEURISM.

### *Femoral and Popliteal Aneurism. External Iliac Artery tied.*

William Smith, aged forty-two, admitted into the Nottingham General Hospital, August 24th, 1824, under the care of Mr. W. Wright, with an aneurism of the femoral artery, at the upper part of the thigh, and another in the ham, both in the left extremity. He gives the following history of them:—About a year

* Surgical Works, vol. ii. p. 225.

